# The importance of determining the amount of ‘therapeutic units’ before using convalescent plasma

**DOI:** 10.2217/fvl-2021-0179

**Published:** 2021-11-04

**Authors:** Yasmine Rangel Vieira, Jorlan Fernandes, Marcelo Alves Pinto, Elba Regina Sampaio de Lemos, Alexandro Guterres

**Affiliations:** ^1^Laboratory of Development Technological in Virology, Instituto Oswaldo Cruz, Fundação Oswaldo Cruz, Rio de Janeiro, RJ, Brazil; ^2^Hantaviruses & Rickettsiosis Laboratory, Instituto Oswaldo Cruz, Fundação Oswaldo Cruz, Rio de Janeiro, RJ, Brazil

**Keywords:** body weight, convalescent plasma, COVID-19, SARS-CoV-2, therapeutic units

To date, apart from supportive care, no specific treatment has been proven to be effective for SARS-CoV-2 infection. To that end, currently, there is a large number of studies to investigate therapies for COVID-19, including convalescent plasma (CP) from recovered COVID-19 patients as treatment. Clinical trials are underway to investigate the efficacy of CP transfusion, new antiviral drugs and vaccines. Although CP has not always proven to be an effective therapy as proven for Ebola virus disease, CP therapy is marked by a success story. Why does this happen? Although some studies have been published or are still in progress, we propose a careful evaluation of the dosage of the therapy, here referred to as ‘therapeutic unit’. The amount of neutralizing antibodies and the period of their administration may determine whether such passive immunotherapy in patients at high risk of deleterious evolution may reduce the frequency of patient deterioration, and thereby mortality.

Administration of CP, serum or hyperimmune immunoglobulin (homologous or heterologous) may be of clinical benefit for the treatment of severe infections of viral etiology. CP therapy has already been used in the treatment of SARS patients, with reports of satisfactory efficacy and safety [1–3]. Attending *in vivo* studies have confirmed the passive transfer of monoclonal antibodies was able to prevent SARS-CoV-2 infection in H2L2 transgenic mice [4]. In addition, studies demonstrate prophylactic and therapeutic effects of using hyperimmune immunoglobulin against SARS-CoV-1 [5] and humanized monoclonal antibodies against SARS-CoV-2 were also able to completely neutralize the infection hamster model [6]. A meta-analysis of 32 human studies of SARS coronavirus infection and severe influenza showed consistent evidence for a reduction in mortality, especially when CP was administered immediately after symptom onset. However, these studies have been frequently classified as low or very low quality works, as they lack control groups and are at moderate or high risk of bias [7]. Regarding Ebola virus disease, the CP therapy was unable to significantly improve patient survival. Griensven and collaborators have evaluated the effectiveness of CP in 84 patients confirmed to have Ebola virus disease in Guinea and had concluded that transfusion of up to 500 ml of CP was not associated with a significant improvement in survival [8].

The US FDA recommends a SARS-CoV-2 neutralizing antibody titer of at least 1:160 as an inclusion criterion for donor selection; or if such a matched unit is not available, that a titer of 1:80 may be considered [9]. Although it is important to guarantee that the donor has sufficient antibody levels, is also (or even more) important to ensure that sufficient titers are transfused to the patient. Effective CP should contain high titers of specific neutralizing antibodies (NAbs) which bind to SARS-CoV-2 and neutralize the viral particles, but variations on antibody titers and characteristics in convalescent plasma donations may occur [10] and must be a concern for clinicians.

In present days, some studies have been reporting the use of CP for COVID-19 treatment [11–13]. For example, Shen and collaborators report findings from a preliminary study of five severely ill COVID-19 patients who were treated using CP from recovered individuals. All patients were in mechanical ventilation due to serious respiratory insufficiency. Although all patients clinical status improved 1 week after CP transfusion, these individuals had also received antiviral treatment with lopinavir/ritonavir and interferon [14]. Therefore, the clinical effect of this CP intervention has not yet been determined, since patients could have recovered due to other treatments administrated in parallel. Patients had received numerous other therapies, including antiviral agents and steroids, making it difficult to distinguish the specific contribution of CP to the clinical course and outcomes [15].

Duan and collaborators have used a single dose of 200 ml of CP from recently recovered donors whose neutralizing antibody titers were above 1:640. This dose was transfused to ten patients as an addition to maximal supportive care and antiviral agents. It is noteworthy that nine of ten (9/10) patients had received arbidol monotherapy or in combination therapy with remdesivir, ribavirin or peramivir, while one patient received ribavirin monotherapy. Additionally, six (6/10) or 60% patients received intravenous methylprednisolone. Treated patients had high NAbs titers before the transfusion; two (2/6) patients had titers of 1:320 and four (4/6) patients had titers of 1:640 [16]. These data, highlight the difficulty of establishing the real role of CP to the clinical course or outcomes of COVID-19. Although cases presented in these reports are relevant, their investigations have important limitations. In the first randomized clinical trial published with COVID-19, Li and collaborators evaluated the efficacy and adverse effects of CP therapy in 52 COVID-19 patients and control group. They used a transfusion dose of CP of approximately 4–13 ml/kg of recipient body weight. Although CP treatment was associated with a negative conversion rate of viral PCR, they found no significant difference in mortality or in the hospitalization time interval from randomization to discharge [17]. Recently, a randomized clinical trial evaluated the efficacy of CP therapy in 160 hospitalized patients; 80 patients received two infusions 48 h apart of 300 ml of CP plus standard of care (SOC) and 80 patients received SOC alone. There was no significant difference between CP+SOC and SOC groups in prespecified secondary outcomes, including 28-day mortality, days alive and free of respiratory support and duration of invasive ventilatory support [13].

Convalescent plasma therapy is marked by a success story. In the mid 1970s, researchers performed a double-blind study of the effects of immune plasma on the treatment of Argentine hemorrhagic fever (AHF). They reported that patients treated with CP had a lower mortality rate compared with subjects treated with ‘normal plasma’, avoiding 90% of deaths [18]. Currently, the AFH is one of the few viral diseases for which there is a specific treatment. For many years, the CP therapeutic dose for AHF was used empirically without knowledge of the NAbs titers present in the units provided. Enria and collaborators determined the therapeutic dose of immune plasma to be given, calculating (in a personalized manner) the ‘therapeutic units’ (TU) of NAbs received by patients, taking into account the patient’s bodyweight, as well as the volume and titer of NAbs in each immune plasma unit provided [19]. Thus, they noted that the mortality rate was 9.09% in patients treated with a dose ranging from 1000 to 2000 TU/kg, 3.70% in patients treated with 2000–3000 TU/kg and that there were no deaths among cases treated with more than 3000 TU/kg. These results show clearly that clinical outcome in cases of AHF treated with CP is correlated with amount of NAbs against Junin virus, the etiological agent of AHF, administered. Thus, they recommended a dose of no less than 3000 TU of NAbs per kg body weight [19].

The impact of CP as a specific treatment is shown in the decrease of the AHF case-fatality rate [20]. This shows that prior titration of NAbs and TU normalization is crucial for clinical trials evaluating use of CP as a viable option for COVID-19 treatment. Depending on the volumes needed and the neutralizing activity of donated CP, preparations could be pooled or individually used [21] before administration, considering NAbs titers and patient weight.

Obese patients have increased risk of aggravation by viral respiratory infections. The link between impaired immune function and obesity raises important questions about the possibility for greater viral pathogenicity in this population. Dietz and Santos-Burgoa highlighted that increased prevalence of obesity in the elderly in Italy compared with China may explain the differences in mortality between the two countries [22]. Duan and colleagues used 200 ml of CP with the neutralizing titers 1:640 in China. In this case, a critically ill patient with a weight of 60 kg would receive 2133 therapeutic units, while a 100 kg patient would receive 1280 TU/kg. Thus, demonstrating the need for an adequate dosage for each critically ill patient. These observations suggest that dose of TU for obese and severely obese patients would likely need an adjustment to be efficient. Unfortunately, to date no study has taken into account the calculation of therapeutic units of NAbs.

Another major problem in COVID-19 is the fast activation of innate immune cells and the change in the levels of many pro-inflammatory effector cytokines, often called a cytokine storm [23]. The CP treatment combined with other therapies such as noninvasive vagus nerve stimulation, can result in an effective treatment of critically ill patients [24]. The vagus nerve stimulation is a potent modulator of immune reactions, suppressing inflammatory cytokine levels [25,26]. The noninvasive vagus nerve stimulation is a neuromodulatory therapy FDA approved for acute and preventive treatment of migraine and cluster headache [24]. The possible antiviral activity found in Li and collaborators studies concerning patients aged 60–80 years with more than 14 days of disease onset is very interesting [17], and makes us wonder if the early administration of CP (within 7 days or less) would improve the therapeutic potential of CP for SARS-CoV-2 infections, as seen for AHF [20].

Although promising, CP still needs to be demonstrated as an effective treatment for COVID-19. Therefore, it is important that studies focus on the ideal dose (TU, as suggested here) by body weight of each critically ill patient. Obese patients with COVID-19 should be carefully managed with prompt and aggressive treatment. In conclusion, the amount of NAbs and the clinical outcome needs to be better evaluated, so therapeutic use of CP for COVID-19 treatment could be compared and better evaluated. Clinical trials taking into account the patient’s body weight and NAbs titers and early administration of CP are necessary in order to improve chances of success.

## References

[1] Cheng Y, Wong R, Soo YOY Use of convalescent plasma therapy in SARS patients in Hong Kong. Eur. J. Clin. Microbiol. Infect. Dis. 24(1), 44–46 (2005). 15616839 10.1007/s10096-004-1271-9 PMC7088355

[2] Hung IFN, To KKW, Lee C-K Convalescent plasma treatment reduced mortality in patients with severe pandemic influenza A (H1N1) 2009 virus infection. Clin. Infect. Dis. 52(4), 447–456 (2011). 21248066 10.1093/cid/ciq106 PMC7531589

[3] Ko J-H, Seok H, Cho SY Challenges of convalescent plasma infusion therapy in Middle East respiratory coronavirus infection: a single centre experience. Antivir. Ther. 23(7), 617–622 (2018). 29923831 10.3851/IMP3243

[4] Fu D, Zhang G, Wang Y Structural basis for SARS-CoV-2 neutralizing antibodies with novel binding epitopes. PLOS Biol. 19(5), e3001209 (2021). 33961621 10.1371/journal.pbio.3001209 PMC8133496

[5] Luo D, Ni B, Zhao G Protection from infection with severe acute respiratory syndrome coronavirus in a Chinese Hamster model by equine neutralizing F(ab′) 2. Viral Immunol. 20(3), 495–502 (2007). 17931120 10.1089/vim.2007.0038

[6] Schäfer A, Muecksch F, Lorenzi JCC Antibody potency, effector function, and combinations in protection and therapy for SARS-CoV-2 infection *in vivo*. J. Exp. Med. 218(3), e20201993 (2021). 33211088 10.1084/jem.20201993 PMC7673958

[7] Mair-Jenkins J, Saavedra-Campos M, Baillie JK The effectiveness of convalescent plasma and hyperimmune immunoglobulin for the treatment of severe acute respiratory infections of viral etiology: a systematic review and exploratory meta-analysis. J. Infect. Dis. 211(1), 80–90 (2015). 25030060 10.1093/infdis/jiu396 PMC4264590

[8] van Griensven J, Edwards T, de Lamballerie X Evaluation of convalescent plasma for Ebola virus disease in Guinea. N. Engl. J. Med. 374(1), 33–42 (2016). 26735992 10.1056/NEJMoa1511812 PMC5856332

[9] Sahu KK, Jindal V, Siddiqui AD, Cerny J, Gerber JM. Convalescent plasma therapy: a passive therapy for an aggressive COVID‐19. J. Med. Virol. 92(11), 2251–2253 (2020). 32437024 10.1002/jmv.26047 PMC7280569

[10] Zhang L, Pang R, Xue X Anti-SARS-CoV-2 virus antibody levels in convalescent plasma of six donors who have recovered from COVID-19. Aging (Albany NY) 12(8), 6536–6542 (2020). 32320384 10.18632/aging.103102 PMC7202482

[11] Joyner M, Wright RS, Fairweather D Early safety indicators of COVID-19 convalescent plasma in 5,000 patients. medRxiv (2020) (Epub ahead of print). 10.1172/JCI140200 PMC7456238 32525844

[12] Salazar E, Perez KK, Ashraf M Treatment of coronavirus disease 2019 (COVID-19) patients with convalescent plasma. Am. J. Pathol. 190(8), 1680–1690 (2020). 32473109 10.1016/j.ajpath.2020.05.014 PMC7251400

[13] Sekine L, Arns B, Fabro BR Convalescent plasma for COVID-19 in hospitalised patients: an open-label, randomised clinical trial. Eur. Respir. J. 2101471 (2021). 34244316 10.1183/13993003.01471-2021 PMC8287736

[14] Shen C, Wang Z, Zhao F Treatment of 5 critically ill patients with COVID-19 with convalescent plasma. JAMA 323(16), 1582 (2020). 32219428 10.1001/jama.2020.4783 PMC7101507

[15] Roback JD, Guarner J. Convalescent plasma to treat COVID-19. JAMA 323(16), 1561 (2020). 32219429 10.1001/jama.2020.4940

[16] Duan K, Liu B, Li C Effectiveness of convalescent plasma therapy in severe COVID-19 patients. Proc. Natl Acad. Sci. 117(17), 9490–9496 (2020). 32253318 10.1073/pnas.2004168117 PMC7196837

[17] Li L, Zhang W, Hu Y Effect of convalescent plasma therapy on time to clinical improvement in patients with severe and life-threatening COVID-19. JAMA 324(5), 460 (2020). 32492084 10.1001/jama.2020.10044 PMC7270883

[18] Maiztegui J, Fernandez N, De Damilano A. Efficacy of immune plasma in treatment of Argentine haemorrhagic fever and association between treatment and a late neurological syndrome. Lancet 314(8154), 1216–1217 (1979). 10.1016/s0140-6736(79)92335-3 92624

[19] Enria D, Fernandez N, Briggiler A, Levis S, Maiztegui J. Importance of dose of neutralising antibodies in treatment of Argentine haemorrhagic fever with immune plasma. Lancet 324(8397), 255–256 (1984). 10.1016/s0140-6736(84)90299-x 6146809

[20] Enria DA, Briggiler AM, Sánchez Z. Treatment of Argentine hemorrhagic fever. Antiviral Res. 78(1), 132–139 (2008). 18054395 10.1016/j.antiviral.2007.10.010 PMC7144853

[21] Brown BL, McCullough J. Treatment for emerging viruses: convalescent plasma and COVID-19. Transfus. Apher. Sci. 59(3), 102790 (2020). 32345485 10.1016/j.transci.2020.102790 PMC7194745

[22] Dietz W, Santos‐Burgoa C. Obesity and its implications for COVID‐19 mortality. Obesity 28(6), 1005–1005 (2020). 32237206 10.1002/oby.22818

[23] Huang C, Wang Y, Li X Clinical features of patients infected with 2019 novel coronavirus in Wuhan, China. Lancet 395(10223), 497–506 (2020). 31986264 10.1016/S0140-6736(20)30183-5 PMC7159299

[24] Staats P, Giannakopoulos G, Blake J, Liebler E, Levy RM. The use of non‐invasive vagus nerve stimulation to treat respiratory symptoms associated with COVID‐19: a theoretical hypothesis and early clinical experience. Neuromodulation Technol. Neural Interface 23(6), 784–788 (2020). 10.1111/ner.13172 PMC7267613 32342609

[25] Tracey KJ. The inflammatory reflex. Nature 420(6917), 853–859 (2002). 12490958 10.1038/nature01321

[26] Borovikova LV, Ivanova S, Zhang M Vagus nerve stimulation attenuates the systemic inflammatory response to endotoxin. Nature 405(6785), 458–462 (2000). 10839541 10.1038/35013070

